# Understanding transporter specificity and the discrete appearance of channel-like gating domains in transporters

**DOI:** 10.3389/fphar.2014.00207

**Published:** 2014-09-12

**Authors:** George Diallinas

**Affiliations:** Faculty of Biology, University of AthensAthens, Greece

**Keywords:** drug transporters, genetic, model systems, *Aspergillus nidulans*, crystal structure, endocytosis/turnover, atypical kinetics

## Abstract

Transporters are ubiquitous proteins mediating the translocation of solutes across cell membranes, a biological process involved in nutrition, signaling, neurotransmission, cell communication and drug uptake or efflux. Similarly to enzymes, most transporters have a single substrate binding-site and thus their activity follows Michaelis-Menten kinetics. Substrate binding elicits a series of structural changes, which produce a transporter conformer open toward the side opposite to the one from where the substrate was originally bound. This mechanism, involving alternate outward- and inward-facing transporter conformers, has gained significant support from structural, genetic, biochemical and biophysical approaches. Most transporters are specific for a given substrate or a group of substrates with similar chemical structure, but substrate specificity and/or affinity can vary dramatically, even among members of a transporter family that show high overall amino acid sequence and structural similarity. The current view is that transporter substrate affinity or specificity is determined by a small number of interactions a given solute can make within a specific binding site. However, genetic, biochemical and *in silico* modeling studies with the purine transporter UapA of the filamentous ascomycete *Aspergillus nidulans* have challenged this dogma. This review highlights results leading to a novel concept, stating that substrate specificity, but also transport kinetics and transporter turnover, are determined by subtle intramolecular interactions between a major substrate binding site and independent outward- or cytoplasmically-facing gating domains, analogous to those present in channels. This concept is supported by recent structural evidence from several, phylogenetically and functionally distinct transporter families. The significance of this concept is discussed in relationship to the role and potential exploitation of transporters in drug action.

## The distinction of channels and transporters

**Figure 1:**
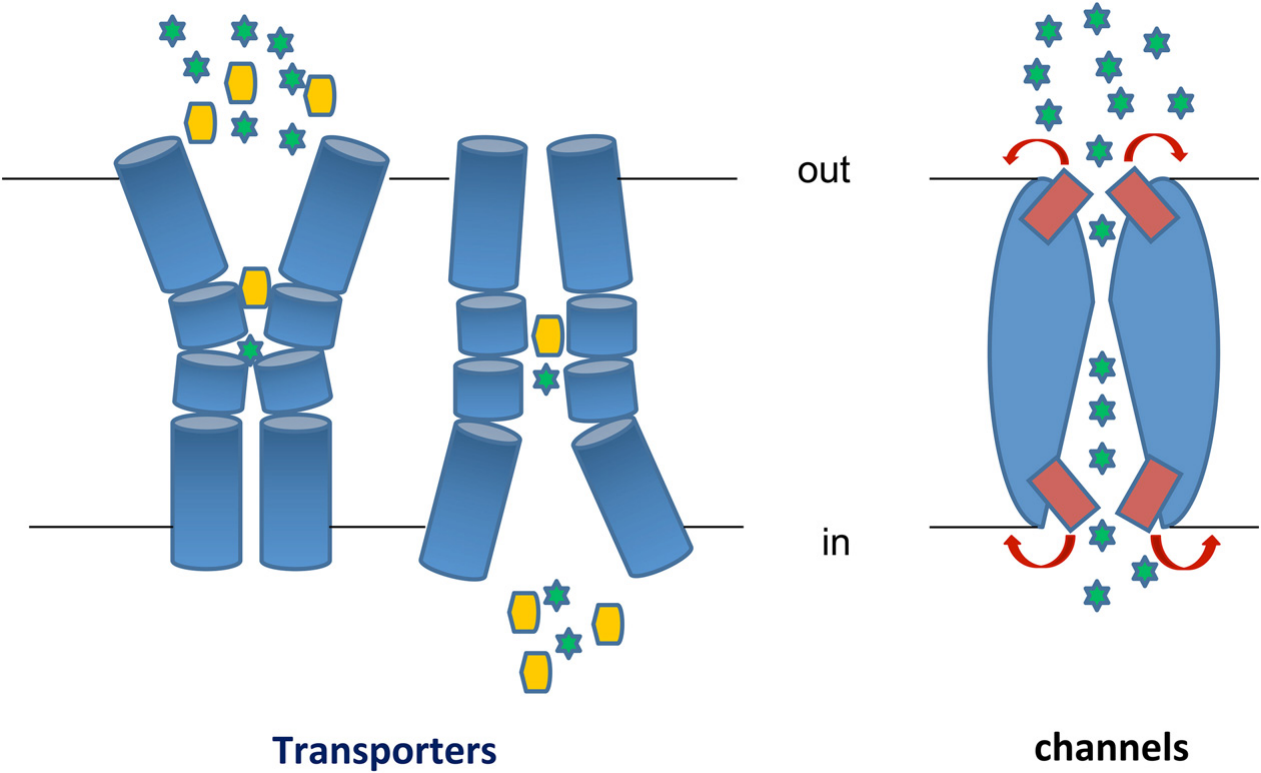
**Transporters vs. Channels. Left:** A transporter undergoes a major “rocking-switch” conformational alteration from an outward- to an inward-facing topology elicited by the binding of a substrate molecule (shown as a yellow hexagon) to specific residues within a substrate-binding pocket located deep in the transporter body at the plane of the plasma membrane. Substrate molecules can be translocated either uphill or downhill of their concentration gradients, depending on the whether the transporter is a secondary active transporter (symporter or antiporter) or a facilitator. The cartoon depicts a symporter, where substrate binding and/or translocation necessitate cation (H^+^ or Na^+^, shown as green stars) binding and transport downhill their concentration gradient. In antiporters, substrate uptake is exchanged with export of another metabolite or cations (e.g., K^+^ or Ca^2+^). During each transport cycle, the bound substrate molecule is never exposed to both sides of the membrane simultaneously. Transporters obey Michaelis-Menten kinetics resembling enzymes, albeit catalyzing a topological rather than a chemical reaction. Most transporters are monomers made of several usually 10–14 transmembrane α-helical, segments (TMS). **Right**: A channel potentially forms a continuous pore but in most cases this pore is restricted by so called selectivity filters and controlled by gating domains or gates (shown in red). Once activated or open, a channel allows continuous translocation of several substrate molecules (shown as green stars), which are mostly ions or small polar compounds, from both sides of the membrane simultaneously. A channel can only mediate facilitated diffusion of substrate molecules down their concentration gradients. Most channels function as homo-oligomers made by 2 or more, mostly α-helical, subunits.

Transporters, permeases, carriers, exchangers, pumps or efflux proteins, facilitators, channels or pores, are all terms used for transmembrane proteins which mediate the transport of solutes, metabolites, drugs, xenobiotics or ions across all kinds of cell membranes, but mostly across the plasma membrane. These terms are rather confusing to the non-specialists, so for the present review, I would like to make a simple but important conceptual distinction between *transporters* and *channels* (depicted in Figure 1). A comprehensive classification system for membrane transport proteins, known as the Transporter Classification (TC) system, analogous to the Enzyme Commission (EC) system, can be found in http://www.tcdb.org/ (Saier et al., 2014). For recent general reviews highlighting differences and similarities in transporters and channels see, Gouaux and Mackinnon (2005), Khalili-Araghi et al. (2009), Krishnamurthy et al. (2009), Conde et al. (2010), Sciara and Mancia (2012).

Transporters (or permeases or carriers) are polytopic transmembrane proteins that function as topological enzymes, that is, they catalyze the translocation of substrates from one side (Greek: *topos*) of the membrane to the other. They comprise a single major substrate-binding site interacting specifically with a single substrate molecule in each transport cycle. Consequently they are characterized, in most cases, with Michaelis-Menten kinetics, with measurable substrate affinity, inhibition or dissociation constants (*K_m_*, *K_i_*, *K_d_*) and transport capacities or rates (*V* or *V_m_*). Substrate binding elicits conformational changes, which eventually produce a transporter conformer open to the opposite side from the one from where the substrate originally bound. In other words, transporters do not contain a continuous open pore accessible from both sides of a membrane. This “rocking-switch” mechanism (Kaback et al., 2011), implicating alternate outward- and inward-facing transporter conformers, has gained significant support from structural, genetic, biochemical or biophysical approaches (see later). Transporters can catalyze the translocation of 10^2^-10^5^ molecules/s and in most cases seem to operate as monomeric functional units made of several (frequently 10–14) transmembrane, mostly α-helical, segments (TMS), well packed between hydrophilic cytoplasmic or extracellular termini. The TMS, which are linked by hydrophilic loops of different lengths depending on the transporter family, host the amino acid residues that interact with substrates or catalyze intra-helical interactions associated with the dynamic translocation of substrates. The hydrophilic loops linking TMS can also play a role in substrate recognition and transport, while in eukaryotes the hydrophilic termini have major roles in the post-translational regulation of transporter trafficking, endocytosis and vacuolar turnover (see later). Transporters can function as facilitators (substrate transport down a concentration gradient), active transporters (substrate transport against a concentration gradient coupled with ATP hydrolysis or most commonly with the symport or antiport of H^+^, Na^+^ or other ions), or exchangers (antiporters) of different substrates translocated in opposite directions. Substrates of transporters can be all kinds of natural metabolites, including lipids, xenobiotics, antibiotics or drugs. In fact, very few natural molecules (e.g., ethanol and some small gases) can efficiently cross the plasma membrane without the need of a transporter. Thus, it becomes apparent that antibiotic and drug action is absolutely dependent on relevant transporters, which can mediate efficient uptake or efflux of these chemicals. However, while we know much on the action of transporter pumping out drugs and leading to multidrug or pleiotropic drug resistance in cancer cells and resistant microbial pathogens (Niero et al., 2014; Paul and Moye-Rowley, 2014; Sun et al., 2014), we know very little on how drugs are taken up by target cells.

Channels (or pores) translocate mostly ions or some small solutes (e.g., water, urea, ammonium or glycerol) and are considered to have a very different mechanism of action compared to transporters, also reflected in a different structure. Similarly to transporters, most channels are made by several, mostly α-helical, TMSs (with the prominent exception of some porins in the bacterial outer membrane which are formed by β-sheets), but in most cases their functional unit is a homo-oligomer made by association of 2 or more subunits. Channels potentially form a continuous pore, commonly in the interphase of different subunits, but in most cases this pore is restricted by so called selectivity filters and controlled by gating domains or *gates*. Selectivity filters are narrow parts of the pore, the size and charge of which is adapted for a specific ion. Gates can exist toward both sides of the membrane and correspond to dynamic domains that open or close in response to a chemical signal. In contrast to transporters, a channel can be open at both sides of a membrane simultaneously. When a channel becomes open one or more ions enter the channel pore and flow rapidly to the other side of the membrane, downstream the concentration gradient(s). Thus, channels function only as facilitators, are much faster (up to 10^8^ ions/s) than transporters, and may lead to the generation of electric currents when translocating charged ions that alter the cellular electrostatic status.

Transporters and channels are ubiquitously involved in cell nutrition, homeostasis, detoxification, stress response, but also in signaling and neurotransmission. Their biological importance is reflected by the observation that all known genomes include at least 5–15% genes coding for transport proteins (http://www.membranetransport.org/). Among the champions of transporters are most free-living bacteria, fungi, and plants, whereas channels are more abundant in metazoa. The importance of transport proteins is also reflected in more than 60 human genetic diseases caused by the malfunctioning of transporters and channels (e.g., Cystic fibrosis, Menkes/Wilsons disease, neurodegeneration, amyotrophic lateral sclerosis, Fanconi-Bickel syndrome, Non-insulin-dependent diabetes, etc.) (http://www.tcdb.org/disease_explore.php). As already mentioned above, transporters, unlike channels, are also key mediators of drug and antibiotic action. The present review has as its main goal to highlight novel findings, somehow neglected in the field of pharmacology, on the mechanism of transporter functioning, which I believe are essential to make a systematic and rational use of transporters as unique tools to develop novel pharmacological approaches.

## Drug action and the potential role of transporters

Most present day drugs are empirically selected based on their efficiency against the primary symptoms of an infection or disease. In the great majority of cases where a drug needs to cross the plasma membrane to exert its activity, we know very little on how the drug enters the cell. Several of the current drugs are hydrophobic compounds that enter the cell, very probably, by slow non-facilitated diffusion through the lipid bilayer of plasma membrane. Some drugs are administered enclosed in liposomes, so that they enter the cell through fusion of liposomes and plasma membranes. Still, a great number of other drugs enter cells through unknown transporters or facilitators (Kell et al., 2011, 2013; Lanthaler et al., 2011). A fact arising from the above observations is that currently used drugs act against target and non-target cells non-specifically. To develop more efficient pharmacological approaches we need, ideally, to use drugs taken up by specific transporters expressed solely in target cells. This becomes a major challenge specifically when the target is a eukaryotic cell, such as cancer cells or fungi and protozoans. Some drugs have been successfully targeted to cancer cells through the action of specific transporters, such as the peptide transporter PepT1 (Nakanishi et al., 1997, 2000) or the glucose transporters GLUT1-5 and SGLT1-3 (Rask-Andersen et al., 2014). However, still most antifungal, antiprotozoan or anticancer drugs used today have not been designed to distinguish between the transporters of target and non-target cells and thus may be taken up non-specifically.

Some present day drugs do not need to cross the plasma membrane to act, but rather target essential plasma membrane components, basically transporters, channels or receptors (Hamman et al., 2007; Kell et al., 2013). Transporters, being the most abundant of these classes of transmembrane proteins, constitute a promising target for specific drug action. ABC-type transporters, which are involved in the efflux of drugs and xenobiotics in cancer cells and resistant microbial pathogens, are among the first to be recognized as ideal drug targets (Choi and Yu, 2014; Niero et al., 2014; Paul and Moye-Rowley, 2014; Sun et al., 2014). However, still little has been achieved in pharmacotherapy through their inhibition. The P-glycoprotein (MDR1), which is the best-studied member of the ABC transporter superfamily, has only recently been exploited for drug development (Zhang and Li, 2013; Saneja et al., 2014). Of the P-type ATPases, both the Na^+^/K^+^-ATPase and the H^+^/K^+^-ATPase are blocked by very useful, and widely used, pharmacological agents (cardiac glycosides and proton pump inhibitors, respectively (Alexander, 2011). Besides ABC efflux proteins, transporters involved in the influx of all kinds of solutes and metabolites (the so called SLC superfamily; He et al., 2009) are also exploited as possible drug targets (Giacomini et al., 2010). Inhibitors of the catecholamine transporters (SLC6A) and Na-K-Cl or Na-Cl symporters (SLC12A) are highly prescribed as antidepressants and diuretics, respectively. The well-studied serotonin (SERT) and dopamine (DAT) transporters are specific targets of several currently used antidepressants (Stahl et al., 2013). The PepT1 transporter involved in oral absorption of di-and tripeptides produced by the digestion of ingested proteins has also become a striking prodrug-designing target recently (Zhang et al., 2013). Still, additional SLC drug targets are currently in clinical trials or under development, for the treatment of a wide variety of diseases and disorders, such as cancer, major depression, Attention Deficit Hyperactivity, osteoporosis, hypertension, cardioprotection, diabetes, constipation and hypercholesterolaemia. Recently, the International Transporter Consortium (ITC) has described seven more transporters of particular relevance to drug development (Giacomini et al., 2010; Huang et al., 2010).

Interestingly however, still most drugs targeting membrane proteins concern receptors, rather than transporters. For example, while G-protein-coupled receptors (GPCRs) represent more than one-quarter of drug targets, currently approved inhibitors of transporters are ten times less (Overington et al., 2006). This contradicts the higher gene number of transporter genes compared to genes encoding “druggable” G protein-coupled receptors (Rask-Andersen et al., 2014). Why is that so? The answer is a dramatic lack of knowledge on the molecular and functional details of transporters. Not only we do not know the structure, function, kinetics or specificity of most human and microbial transporters, but we also know very little on the conditions that affect their expression. Moreover, in order to use transporters either as *gateways* for targeted drug delivery, or as *targets*, we need to know the full complement of transporters in both target and non-target cells.

## Transporters in pathogens as drug gateways or targets

Most bacteria, fungi and protozoa possess hundreds of transporters, some not present in higher eukaryotes (see http://www.membranetransport.org/; Ren et al., 2004 and http://www.tcdb.org/). In addition, even in cases where the host cells and a microbial pathogen possess similar transport activities, these are often characterized by distinct kinetics and specificity profiles. Thus, in principle, transporters of microbial pathogens can well be exploited as specific gateways of antimicrobials. Compatible with this idea is that today, with the easiness and efficiency of genome sequencing and the development of powerful reverse genetics approaches, the systematic characterization of the complete complement of transporters in the most important pathogens is only a matter of dedicated effort. In such an effort, and especially to carry out sophisticated reverse genetic approaches and rigorous biochemical assays, the use of model bacteria, fungi and protozoa, will constitute a major step. This is necessary as one of the major problems studying the function and specificity of a given transporter is the existence of a multitude of transporters of overlapping specificities in most organisms. Thus, it is practically impossible to rigorously characterize with simple uptake assays the kinetics and specificity of a transporter if other similar transporters are also present. This problem can only be overcome by studying a single transporter in an appropriate genetic background lacking any other functionally related transporter. Such strains can be easily constructed and tested by combining multiple gene knock-out mutations in model microbes such as *Escherichia coli*, *Aspergillus nidulans* or *Saccharomyces cerevisiae*. Transporters from pathogenic microbes can also be isolated, expressed and functionally characterized in appropriate strains of model relatives, as for example has been the case for a number of *A. fumigatus* and *Candida albicans* purine transporters studied in *A. nidulans* or *S. cerevisiae* (examples in Goudela et al., 2006, 2008; Keniya et al., 2013), or some protozoan transporters studied in *S. cerevisiae* (Burchmore et al., 2003; Natto et al., 2005; Al-Salabi et al., 2007; Papageorgiou et al., 2008b).

Once transporters of model or pathogenic microorganisms are genetically, biochemically and physiologically characterized, the next step will be to identify the most promising among them in respect to antimicrobial drug recognition and uptake. Selected transporters based on their specificity profiles and preliminary *in vivo* assays can be tested for the translocation of existing powerful antimicrobials or novel drugs, which can be designed or screened from chemical libraries through rational or semi-rational approaches (Zhu et al., 2013). These later approaches will necessitate deep knowledge on the molecular details underlying transporter-substrate interactions and the mechanisms of substrate translocation. Such knowledge should be obtained through crystal structures, homology modeling, docking approaches and Molecular Dynamics (MD), but also by extensive mutational and biochemical analyses uniquely feasible in model microorganisms.

An alternative to the exploitation of microbial transporters as drug gateways is their use as drug targets in cases where transporters prove essential for survival or virulence. Recently, such a case was exemplified by *Borrelia burgdorferi*, the causative agent of Lyme disease (Samuels and Radolf, 2010; Jain et al., 2012). Borrelia is transmitted to humans by the bite of infected ticks belonging to a few species of the genus Ixodes. The infection is usually eliminated by antibiotics, only if treated early but some individuals do not respond to antibiotic treatment. Moreover, present day vaccines present autoimmune side effects and are expensive. An alternative highly targeted pharmacological approach can now be developed based on the fact that *B. burgdorferi* lacks the enzymes required for *de novo* synthesis of purines and therefore its life cycle and virulence depends on two purine transporters of the NAT family (see later), named BBB22 and BBB23. This is supported by the observation that *B. burgdorferi* lacking *bbb22-23* was non-infectious in mice (Jain et al., 2012). Thus, any compound specifically inhibiting the BBB22/23 transporters might potentially serve as a highly specific drug for treating Lyme disease. Importantly, the fact that BBB22/23 transporters belong to a NAT subfamily, called AzgA (Cecchetto et al., 2004; Krypotou et al., 2014), absent in humans, enhances the prediction that BBB22/23-specific drugs might well not be associated with undesirable side effects.

## Approaching transporter structure-function relationships

Until some years ago, most of what we knew on the function of transporters has come through genetic and biochemical approaches mostly performed with the lactose permease of *E. coli* (LacY) and a handful of other transporters, the majority of which were of bacterial origin (for LacY reviews see Kaback et al., 1993, 1994, 2001; Kaback and Wu, 1997). The reason for this is directly related to the low expression level, hydrophobic nature, big size and transmembrane topology of transporters. Even today, transporters cannot be studied by biophysical or structural approaches in their natural membrane environment. The use of detergents during purification for crystallographic or other biophysical methods, or reconstitution in proteoliposomes for functional assays, do not take into account the significant role of specific lipid species in transporter folding, functioning or turnover. This problem becomes even more serious when it concerns eukaryotic transporters, where lipids play additional regulatory roles related to membrane trafficking and PM expression of a transporter.

Because of these technical problems, Kaback working on LacY, and a handful of other scientists studying other transporters, initiated rational genetic and biochemical approaches, combined with simple radiolabeled substrate uptake assays in intact bacteria or other easily manipulated cells, such as fungi, protozoa, red blood cells, oocytes or cultured mammalian cell lines. The work with LacY is by far the most impressive (see reviews Kaback et al., 1993, 1994, 1997). Each of the 417 aminoacyl side-chains in LacY has been mutated and remarkably, fewer than 10 residues located in TMS4, TMS5, TMS7, TMS8, TMS9, and TMS10, were shown to be irreplaceable for active lactose transport (Frillingos et al., 1998). The employment of Cys-scanning mutagenesis and its downstream applications have proved to be a breakthrough tool in assigning functional or structural roles to specific residues in transporters (Frillingos et al., 1998). Thus, even before the appearance of the first crystal structure of any transporter in early 2000s, Kaback and co-workers have proposed a theoretical model of the topological arrangement of transmembrane domains in LacY, provided rigorous evidence on which amino acid residues are involved in substrate and H^+^ binding and translocation, and proposed mechanism for transport catalysis.

In this mechanism, the substrate and the H^+^ ion interact within a single binding pocket accessible in an outward-facing conformation of the transporter, and this binding subsequently promotes a dramatic conformational change leading to a cytoplasm-facing transporter capable of liberating the substrate and the H^+^ ion into the cell. This “rocker-switch” mechanism, which implies that transporters are never accessible to substrates from both sides of the membrane, but they rather alternate between two conformers, has been subsequently supported by numerous kinetic, biochemical and biophysical studies not only for LacY (Abramson et al., 2004; Kaback et al., 2011), but also for several other transporters (Forrest et al., 2011; Shi, 2013; Penmatsa and Gouaux, 2014; see also later).

The “rocker-switch” mechanism predicts that mutations affecting substrate binding affinities or substrate specificity, should affect residues interacting with substrates directly or indirectly. Mutational and second-site suppressor analyses have supported that idea in general (Cain et al., 2000; Johnson et al., 2001; Green et al., 2003). In some specific cases however, mutations modifying transporter specificity in LacY (Naftalin, 2010) or other transporters (see later) could not fit with the simple “rocker-switch” alternating model. This issue will be discussed in the next section of this review.

In recent years (since 2003), the availability of 2D and 3D structures obtained by EM crystallography and X-ray, as well as contributions from computational and theoretical approaches, have greatly enhanced our understanding of transporter structure-function relationships. The first crystal structure of a secondary active transporter at atomic resolution was obtained in 2002 (bacterial multidrug efflux transporter AcrB; Murakami et al., 2002), while LacY was crystallized in 2003 (Abramson et al., 2003). At present there are <50 transporter structures analyzed at atomic levels of 1.5–3.7 Ȧ in total (see http://blanco.biomol.uci.edu/mpstruc/). Among those, less than 20 concern ATP-dependent ABC primary active transporters and the rest are cation symporters, facilitators, antiporters or exchangers. The great majority of transporters consist of 10–14 α-helical transmembrane segments (TMS) connected via intracellular and extracellular loops, and cytoplasmic N- and C-terminal regions. These transporters seem to function as monomers, although some also form dimers or oligomers. Shorter transporters made of 3–6 TMS seem to form homo- or hetero-oligomeric functional complexes. Surprisingly, transporters of functionally and evolutionary distinct protein families with no primary amino acid sequence similarity and different substrate specificities seem to exhibit similar folds.

The majority of known transporter structures fall within two types of folds; the 6+6 of Major Facilitator Superfamily (MFS) type (Abramson et al., 2004; Kaback et al., 2011; Madej et al., 2014), and the 5+5 intertwined LeuT/Mhp1 type (Yamashita et al., 2005; Weyand et al., 2008; Forrest et al., 2011; Shi, 2013; Penmatsa and Gouaux, 2014). 6+6 or 5+5 depicts the α-helical transmembrane segments present in each type of transporter fold. A variation of the 5+5 fold is exemplified by the 7+7 transporters of the NAT/NCS2 family (Lu et al., 2011; Västermark and Saier, 2014). In fact, several of the 5+5 transporters contain 2 or 4 extra helices, having in total 12 or 14 TMS, but these extra domains are probably involved in oligomerization, trafficking or turnover, rather than transport activity (Västermark and Saier, 2014). The common structural folds found in symporters, facilitators and antiporters strongly suggest that overall protein architecture does not dictate the mode of transport. Despite important structural differences, as for example the site of the major substrate binding site and the mechanism of transport, all types share some common themes, such as an apparent two-fold internal structural symmetry, or the presence of discontinuous membrane α-helices hosting residues involved in substrate and/or ion binding, suggesting some common ancestral origins (Forrest et al., 2011; Krishnamurthy and Gouaux, 2012; Shi, 2013; Penmatsa and Gouaux, 2014; Västermark and Saier, 2014).

How has the recent boom in transporter crystallography changed our view on transporter structure-function relationships and the mechanism of transport catalysis? Do residues found to be responsible for substrate binding or translocation by purely genetic and biochemical analyses agree with their position in the crystal structures? Or *vice versa*, do the models proposed account for the effects produced by mutation of specific residues? The answer is generally yes, since in most cases genetics and biochemistry proved to be in good agreement with structural studies. A more difficult question to be answered is whether the alternating conformation “rocker-switch” mechanism is still valid? The answer to this question is a little more complex and is discussed below.

**Figure 2:**
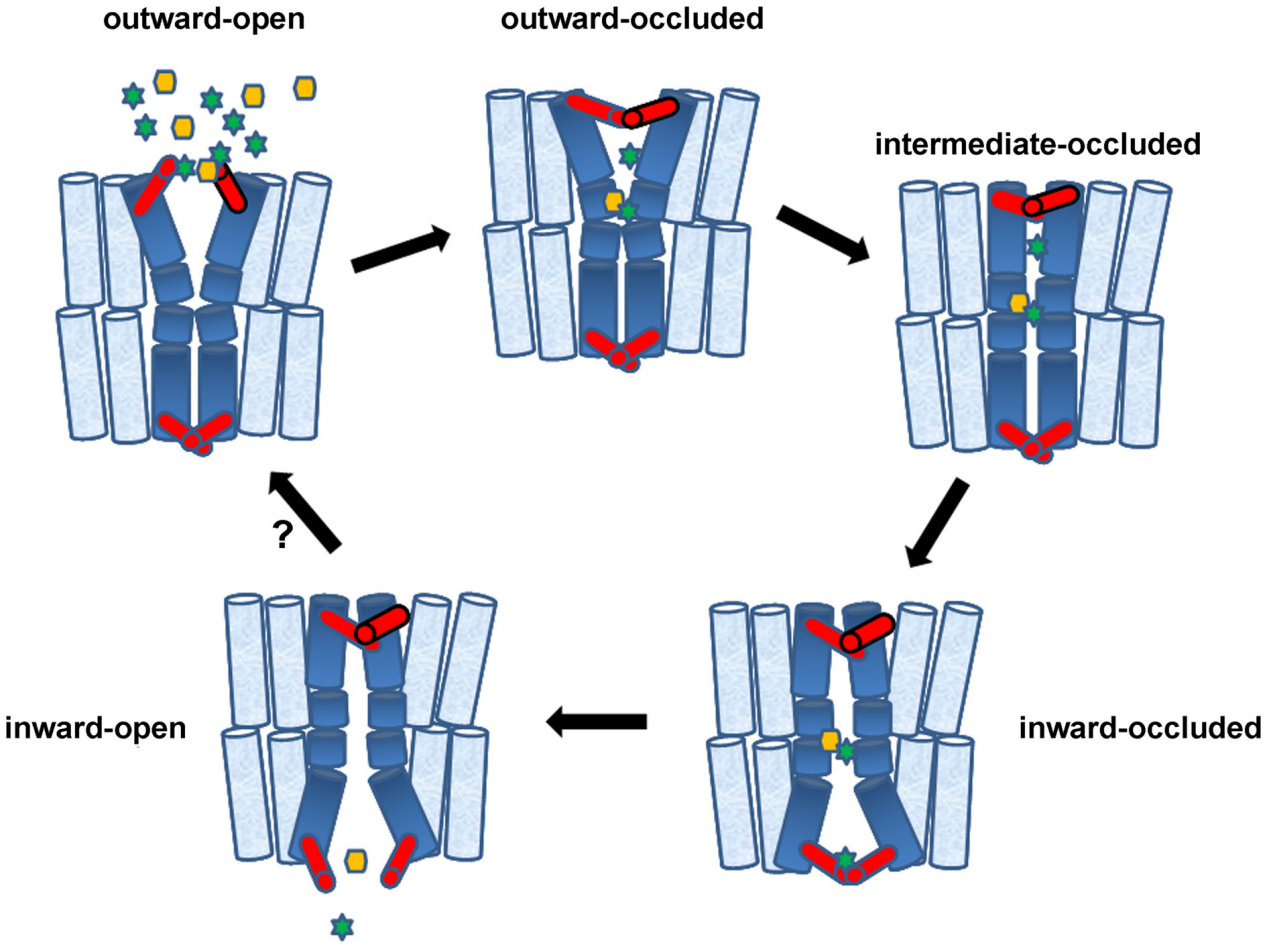
**Transporters in action and the role of gates**. Accumulating evidence supports a theoretical trailer of alternating transporter conformations involving five structurally distinct basic conformations: (1) outward-facing with extracellular gate open (cytoplasmic gate occluded), (2) outward-facing with extracellular gate occluded (cytoplasmic gate remains occluded), (3) intermediate with both gates occluded, (4) inward-facing with gates occluded, (5) inward-facing with only cytoplasmic gate open. Gates, shown in red, can be made by a few flexible amino acids or bigger domains made of parts of α-helices. Starting from (1), binding of Na^+^ or H^+^ (green stars) at the extracellular gate or deeper into the major substrate-binding pocket, stabilizes the outward-facing open state, creating a high-affinity substrate-binding site deep in the transporter body. Substrates (yellow hexagon) might also bind selectively to the extracellular gate. Following substrate and potentially additional ion binding, the extracellular gate closes and this closure promotes an induced-fit transition to the intermediate or inward-facing occluded state. This transition involves major conformational movements of flexible α-helices or parts of helices (shown in deep blue). In the inward-facing state, release of Na^+^ or H^+^ stabilizes the opening of the intracellular gate and triggers substrate release. The empty inward-facing open transporter can then transit back outward-facing state and, thereby, complete the transport cycle. The diagram shows a single-step transition from “inward-open” to “outward-open,” whereas this might take several intermediate steps. The cartoon depicts a symporter but the same model can be easily adapted to facilitators and antiporters. This model essentially combines aspects of the rocker-switch model with channel-like gating. Genetic, biophysical and computational approaches suggest that gates can act as secondary transient binding sites of substrates and ions.

What the recent transporter structures tell us is that transporters can be found in a series of interrelated intermediate conformations and not simply in outward- or inward-facing topologies. In other words, according to the current model based on the available structures, a transporter cycles through a set of defined conformational states providing a unique structural framework necessary for efficient substrate transport. The principle of a transport cycle is illustrated in Figure 2. In this model, the substrate(s) first binds to the empty transporter in the outward facing conformation, where the binding site is only accessible from the outer side. This is followed by the *closure* (or *occlusion*) of outer molecular *gates* to hinder substrate diffusion. The gate closure is facilitated by the substrate-induced rearrangement of single amino acid side chains or by the bending of single α-helices, parts of helices or helical hairpins. The transport cycle then proceeds by a substantial conformational change from the occluded (closed) outward-facing to the occluded inward-facing conformation. During this structural switch the transporter passes through the intermediate occluded form, where the substrates are inaccessibly buried within the protein. This is followed by the opening of the inner molecular gates thus enabling the release of the substrates from the transporter protein into the cytosol. The transport cycle is completed when the transporter switches from the empty internal form, back to the empty external conformation, enabling the protein to start a new cycle (Diallinas, 2008; Forrest and Rudnick, 2009; Reyes et al., 2009; Forrest et al., 2011; Madej et al., 2012, 2014). In the case of an antiporter, a single substrate is transported during the conversion from the occluded outward-facing to the occluded inward-facing conformation, while the co-substrate is transported in the returning step from the inward to the outward conformation. In summary, the current idea is that transporters function through a cascade of conformational alterations, some of which are “small” (gate closure and opening), some more dramatic (outward to inward transition of the main translocation trajectory). A rocker-switch alternating access mechanism, at its basic principles, is thus still true for both the 6+6 and 5+5/7+7 type transporters.

What is drastically new in this modified conformation-switch mechanism is the presence of functionally and topologically independent channel-like gates controlling the selective access of substrates to the major transport binding site. Thus gates made a slow and discrete appearance in transporters. This might have been a surprise to most structural biologists, but not to geneticists. In 2001, long before crystal structures revealed the presence of selective gates, their existence has been proposed through the functional characterization of mutations affecting the specificity of a fungal purine transporter. Unfortunately, in our days, structural biologists or hardcore biochemists seem to “miss” information coming from simple genetic model systems. This review wishes to highlight how genetic approaches can be used to establish the existence of gates in transporters and study their role in substrate selection, but, unexpectedly, also in transporter turnover.

## Classical genetics predicted the existence of gates and shed new light on the molecular basis of transporter specificity

UapA is one the best functionally characterized eukaryotic transporters (reviewed in Diallinas and Gournas, 2008; Gournas et al., 2008). It is a high affinity, high-capacity, H^+^ symporter specific for the uptake of uric acid or xanthine in *A. nidulans*. UapA has also moderate affinities for some xanthine or uric acid analogs (e.g., 2- or 6-thio analogs or 3-methylxanthine), including the drugs allopurinol or oxypurinol (Goudela et al., 2005). The *uapA* gene was identified in the mid 60 s through mutations leading to resistance to a morphological effect produced by 2-thioxanthine (Darlington and Scazzocchio, 1967). Mutations in the promoter of *uapA*, were probably among the first *cis*-acting regulatory mutations isolated and studied in a eukaryote (Arst and Scazzocchio, 1975). The gene was cloned in 1989 by insertional inactivation and sequenced early in the 90 s (Diallinas and Scazzocchio, 1989; Gorfinkiel et al., 1993). Since then, UapA has been extensively studied in respect to transcription and post-translational regulation in response to physiological signals, conidiospore germination or asexual development. In summary, the transcription of the UapA is developmentally induced early during germination (Amillis et al., 2004), whereas in mycelia transcript levels are highly regulated in response to substrate-induction and ammonium repression (Diallinas and Scazzocchio, 1989; Gorfinkiel et al., 1993). Furthermore, plasma membrane UapA is finely down-regulated by rapid ubiquitination, endocytosis and vacuolar turnover, in response to ammonium (Pantazopoulou et al., 2007; Gournas et al., 2010) or excess substrates (Gournas et al., 2010; Karachaliou et al., 2013) Interestingly, substrate-elicited endocytosis, unlike ammonium triggered internalization, depends on the transport activity of UapA (see later).

**Figure 3:**
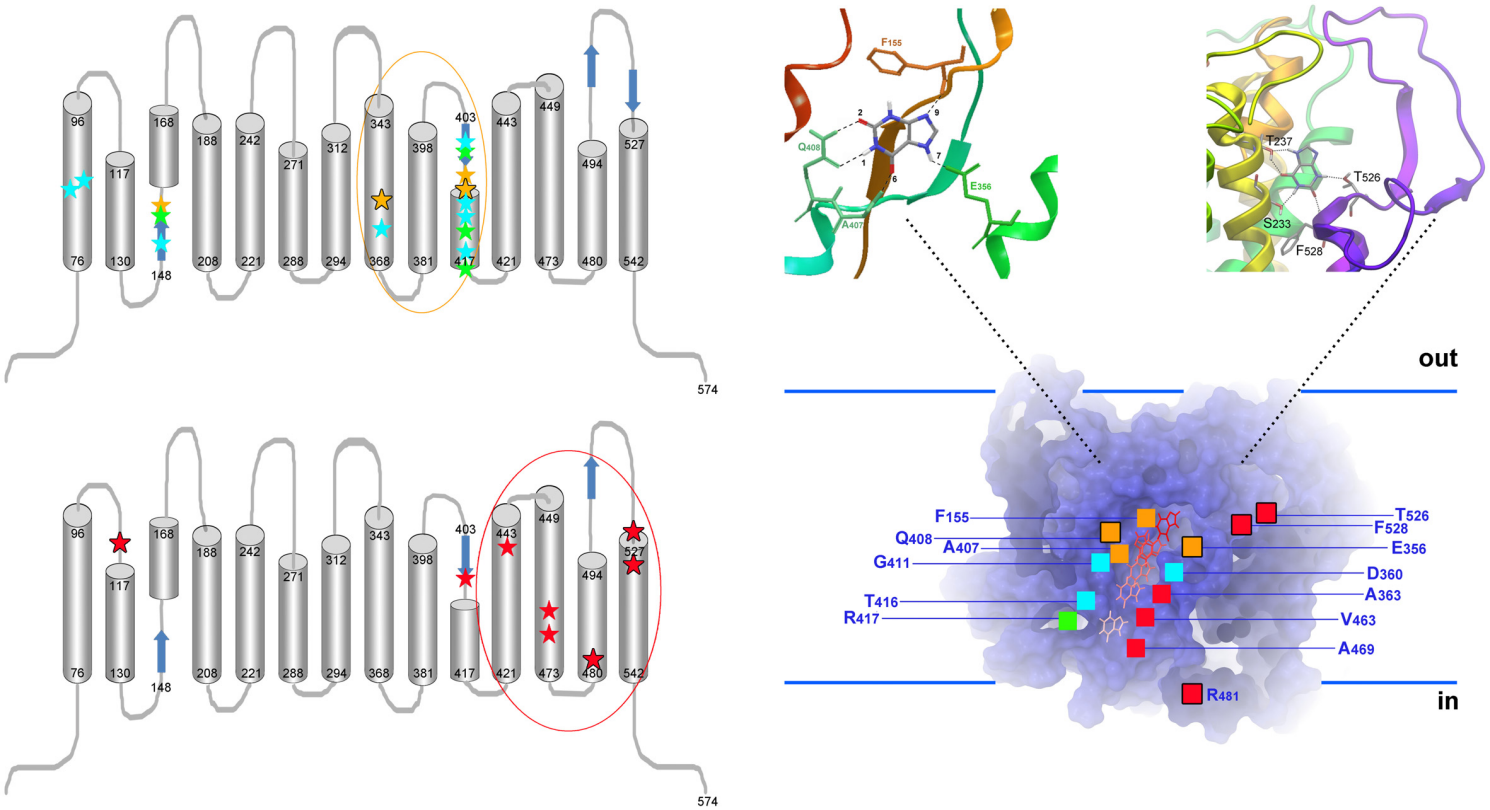
**Topology of UapA residues critical for function and specificity. Left, upper panel:** Topology of residues critical for the function and transport kinetics of UapA. All important residues map in TMS1, TMS3, TMS8, and TMS10. Residues interacting directly with substrate (xanthine) are highlighted as yellow stars (Ser155, Glu356, A407, and Gln408). Among those, Glu356 and Gln408 interact with substrates through their side chains (further highlighted with a black outline). These two residues are irreplaceable for function. In green are residues (Ser154, Phe406, G411, Arg417) affecting the local topology of neighboring substrate-binding residues. Mutations in these residues can differentially affect the affinity for different substrates (e.g., uric acid vs. xanthine). In blue are residues (Gln85, His86, Met151, Asp360, Thr405, Asn409, Asn410, and Thr416) involved in dynamic interdomain interactions between helices TMS1, TMS3, and TMS10 (e.g., Gln86-Thr416, His86-Asn409, His86-Met151, Asp360-Asn410-Thr416). Mutations in these residues, which are often cryosensitive, abolish or dramatically reduce transport activity without affecting significantly substrate binding. Little is known on the residues binding H^+^ ions, but a Glu356 and Asp360 are possible candidates. Evidence for the above conclusions comes from functional assays of a plethora of mutants using a set of >25 different purines or purine related analogs, as well as, docking and MD approaches. Among mutations concerning residues critical for determining the affinity (K*_m/i_* values) for physiological substrates and ligands, only Gln408Glu shifts significantly the *binding* specificity of UapA toward novel ligands (hypoxanthine, guanine, uracil). Importantly however, even in this case, the mutated UapA cannot transport the novel ligands. In other, words no mutation affecting the functioning of the presumed substrate binding site modifies dramatically the specificity of UapA. **Left, lower panel:** Topology of residues critical for determining the substrate specificity of UapA. All important residues map in TMS11 (Ala441), TMS12 (Val463, Ala469), TMS13 (Arg481), TMS14 (T526, Phe528) and in the loop linking TMS1-TMS2 (Gln113). In other words, all specificity important residues are located in domains distinct from those involved in the functioning of the major substrate binding site. A single exception is Phe406, which is located in TMS10. The importance of these residues was recognized through the functional analysis of randomly, directly or indirectly, selected mutants able to grow on non-physiological UapA substrates (e.g., hypoxanthine or adenine). Most critically, no mutation in the residues affecting UapA specificity has a significant effect on the *K_m_* and *V_m_* values of UapA for its physiological substrates, strongly supporting the idea that none of these mutations concerns a residue located in the major substrate-binding site. Among specificity mutations, the most prominent effects were obtained with mutations at Arg481, Thr526, and Phe528, highlighted with a black outline (Amillis et al., 2001; Vlanti et al., 2006; Papageorgiou et al., 2008a; Kosti et al., 2010). **Right:** Homology model of UapA built using the inward-facing crystal structure of the bacterial UraA permease. The model shows the major substrate binding site and a translocation pathway leading to the inside of the cell (Kosti et al., 2012). The substrate (e.g., xanthine) binding position, located *in silico* through docking and MD approaches, is highlighted in the insert on the top left. The insert on the top right depicts a putative secondary binding site, coincident with residues Thr526 and Phe528, located at an outward presumed gate, as this was defined by genetic, docking and MD approaches. A speculative inward-facing gate is also supported by mutations in Arg481. Combinations of different gate mutations (e.g., Arg481Gly/Thr526Leu or Arg481Gly/Phe528Ser), or gate and major binding site mutations (e.g., Arg481Gly/Gln408Glu, Thr526Leu/Gln408Glu, Thr526Met/Gln408Glu, Phe528Ala/Gln408Glu, Phe528Ser/Phe406Ala), lead to UapA versions with distinct transport activities and specificities, supporting the functional independence of the two presumed gates and the major substrate binding site (Papageorgiou et al., 2008a; Kosti et al., 2010, 2012; Amillis et al., 2011).

In the recent years novel tools have been designed that allowed the direct and rigorous study of the function of UapA. These tools were based on four important technical innovations: (i) the development of rapid uptake assays using germinated conidiospores of *A. nidulans*, which avoided technical difficulties associated with the use of mycelia (Krypotou and Diallinas, 2014), (ii) the genetic identification of all major *A. nidulans* transporters involved in purine, pyrimidine or nucleoside transport, which eventually led to the construction of appropriate null strains, permitting the study of a specific transporter in a “clean” genetic background devoid of similar transporters (Pantazopoulou and Diallinas, 2006; Diallinas, 2008; Krypotou and Diallinas, 2014), (iii) the development of very efficient reverse genetic approaches in *A. nidulans*, which permitted the rigorous study of any mutant transporter version expressed from it genetic locus (Nayak et al., 2006; Szewczyk et al., 2006), and (iv) the employment of *in vivo* fluorescent microscopy approaches using GFP- or RFP-tagged functional transporters, which directly allowed the classification of transporter mutants in those affected in folding, trafficking or transport function *per se* (Valdez-Taubas et al., 2004; Vlanti et al., 2006; Pantazopoulou et al., 2007). Using basically these four tools, dozens of UapA mutations or chimeric molecules have been characterized at the level of protein expression, stability, sorting and turnover, as well as, of function and specificity. In addition, using standard transport competition assays with a plethora of purine or pyrimidine analogs, an extended substrate binding specificity profile of UapA has been obtained. These purely genetic, biochemical and kinetic approaches led to a number of significant conclusions concerning specific amino acid residues that are involved in substrate binding or/and transport (see Figure 3). Among functionally important amino acid residues, Glu356 and Gln408 were proposed to interact directly with UapA substrates through H bonding implicating their polar side chains, whereas seven more residues proved critical for substrate translocation (see legend of Figure 3 and Diallinas et al., 1998; Meintanis et al., 2000; Koukaki et al., 2005; Papageorgiou et al., 2008a). These conclusions were strongly supported when the first modeled structure of UapA became feasible (Amillis et al., 2011; Kosti et al., 2012), immediately after the publication of the crystal structure of a bacterial homolog, namely the UraA uracil permease of *E. coli* (Lu et al., 2011). The structural model of UapA and subsequent docking and MD approaches showed that a major xanthine binding site is formed by four specific residues in TMS3 (Phe155), TMS8 (Glu356) and TMS10 (Ala407 and Gln 408). The same residues were shown to bind xanthine in the homologous *E. coli* XanQ permease (Karatza et al., 2006; Karena and Frillingos, 2009, 2011; Georgopoulou et al., 2010; Mermelekas et al., 2010). Importantly, the work on UapA has shown that reverse genetics and biochemical approaches in a eukaryotic model system, similarly to work performed in *E. coli*, constitute unique tools not only for understanding the function of transporters, but also to validate structural models emerging from homology modeling approaches with available crystal structures.

Interestingly, in the course of extensive genetic and biochemical analyses of rationally designed mutants of UapA, no mutant was obtained that altered significantly the specificity of UapA. In other words, none of the UapA mutants studied acquired the capacity to transport other nucleobases, such as adenine, guanine, hypoxanthine or pyrimidines, which are all structurally similar to the transporter physiological substrates, xanthine or uric acid. This somehow contradicted the observation that several among the functional mutations did modify the affinity for xanthine, uric acid or some of their analogs, suggesting that these mutations have modified the substrate binding site of UapA. The single exception among the mutations characterized in respect to UapA substrate specificity was Gln408Glu, a mutation replacing a residue proposed to interact directly with substrates. Gln408Glu was a crysosenstive mutation which at the permissive temperature led to moderate binding of hypoxanthine or guanine, but still could not lead to detectable UapA-mediated transport of these purines (Meintanis et al., 2000; Koukaki et al., 2005). The lack of a UapA mutant capable of binding *and* transporting hypoxanthine, or any other purine that is not uric acid and xanthine, was a surprise. Hypoxanthine is smaller than xanthine and very similar in structure, so how does UapA select between these two purines? Or how can UapA distinguish between allopurinol and hypoxanthine in favor of the former (Diallinas, 2013), given that allopurinol and hypoxanthine are also very similar in structure? These questions already suggested that transporter specificity might not simply be determined by interactions within a freely accessible major substrate binding site, but might rather depend on complex inter-domain synergistic interactions, which probably underlie a transport mechanism more sophisticated than that described in the originally proposed rocker-switch model.

In an attempt to suppress the cryosensitivity of the Gln408Glu mutant, a second-site suppressor, Phe528Ser, was identified within TMS14 of UapA (Amillis et al., 2001; note that in the original article UapA was thought to have 12 TMSs, so Phe528Ser was originally placed in TMS12). Subsequent kinetic analysis of systematic site-directed replacements of Phe528 revealed that substitution of Phe with small, aliphatic, amino acids led to transporter molecules that could catalyze the transport of a series of purines, albeit with low affinity (>1 mM), without affecting the high-affinity (5–10 μM) for the physiological substrates, uric acid and xanthine (Vlanti et al., 2006). Subsequently, a different genetic screen designed to obtain UapA mutants able to transport adenine, revealed two more residues that could affect UapA specificity in a similar way (Papageorgiou et al., 2008a). These residues were the partially conserved Thr526 and the variable Gln113, the first lying in TMS14 and the second in the putative extracellular loop between TMS1 and TMS2. Finally, a different direct selection scheme, using as the starting strain Phe528Ser, led to the isolation of more mutants which could transport even more efficiently hypoxanthine or adenine, but again the relevant mutation had no significant effect on the kinetics of transport of xanthine or uric acid (Kosti et al., 2010). The relevant mutations concerned residues in TMS11 (Ala441), TMS12 (Val463, Ala469), and TMS13 (R481). The position of functionally critical residues in UapA is shown in Figure 3.

Thus, random direct or indirect genetic screens led to the identification of several specificity mutations in UapA domains (TMS1-TMS2 loop, TMS11, TMS12, TMS13, and TMS14) none of which is part of the presumed substrate binding pocket, which is made by residues in TMS3, TMS8, and TMS10. All these specificity mutations enlarged the capacity of UapA to transport purines, pyrimidines and bulky analogs of xanthine. Most critically, none of the specificity mutations affected significantly the *K_m_* values of UapA for its natural substrates xanthine or uric acid. This last observation dismissed the possibility that the specificity mutations have affected the basic architecture of the UapA substrate-binding site. If they did, they would be expected to affect the affinity constants for uric acid or xanthine, as well. Based on this logic, we have proposed that specificity mutations should map in domains acting as *selective filters* of channel-like *gates*. In other words, UapA, and possibly other transporters, seems to contain selective genetically distinguishable outward- and cytoplasm-facing gates that control the access or removal of substrates from a major substrate binding pocket. Specificity mutations very probably loosen the selective role of these gates, so that, non-physiological substrates can “leak” toward or out of the major binding site. This idea predicted that we might be able to convert UapA into a very efficient hypoxanthine transporter if we combine specificity mutations in the gates with mutations in the *bona fidae* substrate binding pocket, or combine gate mutations that simultaneously loosen the outward and inward-facing gates. This was in fact the case. Combinations of different gate mutations (e.g., Arg481Gly/Thr526Leu or Arg481Gly/Phe528Ser), or gate and major binding site mutations (e.g., Arg481Gly/Gln408Glu, Thr526Leu/Gln408Glu, Thr526Met/Gln408Glu, Phe528Ala/Gln408Glu, Phe528Ser/Phe406Ala), led to UapA versions with distinct transport activities and specificities, supporting the functional independence of the two presumed gates and the major substrate binding site (see Figure 3 and Papageorgiou et al., 2008a; Kosti et al., 2010).

What is the lesson from the classical genetic approaches used in UapA? No rational design would have predicted the role of the presumed gate residues which affect substrate specificity as these, unlike residues in the major binding site, are not well conserved. Why are these residues not well conserved? In fact, in the case of the NAT (Nucleobase Ascorbate Transporters) family where UapA belongs, there is no genetic evidence supporting the existence of residues critical for substrate specificity in analogous domains of the bacterial homologs. For example, in XanQ (xanthine permease), mutations in Asn430 and Ile432, which correspond to residues Thr526 and Phe528 in UapA, do not confer the ability for transport of purines other than xanthine, in sharp contrast with the case of the *A. nidulans* transporter (Papakostas et al., 2008; Frillingos, 2012). The analogous XanQ mutations simply moderately affect the affinity of xanthine binding and allow the transport of some xanthine analogs with bulky groups. This in turn might suggest that in XanQ the domain analogous to gate domain in fungi (*A. nidulans*) seems to function as a substrate filter rather than a selectivity gate.

Overall, it seems that the evolution of substrate selectivity gates is a functional novelty that might have arisen independently in different transporter families or in different members of the same family. The reason for that might be connected with a specific selective advantage related to the need for more dynamic control of substrate binding and transport in eukaryotic cells (see also later). The variability in the evolution of gating mechanisms is most strongly supported by recent structural reports that show that the closure and opening of gates seems to be elicited by different mechanisms and distinct molecular elements in different transporters (see below). Finally, the evolution of gates seems to have also affected how transporters are regulated at the level of cellular expression, sorting, endocytosis and turnover, as will be discussed later.

## Are gates in transporters substrate-selective?

Accumulating evidence mainly from crystal structures, but also from biophysical (e.g., interspin-distance measurements from double electron-electron Resonance, distance-dependent quenching of Trp fluorescence), biochemical (site-directed alkylation) and homology modeling approaches, supports directly the existence of transiently *open* and *occluded* conformations of transporters (Smirnova et al., 2007; Fang et al., 2009; Gao et al., 2009; Shaikh and Tajkhorshid, 2010; Forrest et al., 2011; Kaback et al., 2011; Zhao and Noskov, 2011; Jiang et al., 2012; Krishnamurthy and Gouaux, 2012; Madej et al., 2012; Shi, 2013; Kumar et al., 2014; Penmatsa and Gouaux, 2014; Stelzl et al., 2014; Valdés et al., 2014). In the analyzed crystal structures (see Table 1), the open states face either the cytoplasm or the extracellular space, and in both cases seem to allow free access of substrates and ions into a single major binding site, found relatively deep in the transporter body. In some crystal structures substrates or ligands and inhibitors have been found bound to partially open conformations of transporters, whereas in other transporters are “caught” empty. In the fully occluded state, the major substrate and ion binding site is occupied and seems inaccessible from either the cytoplasm or the extracellular side. This is so because specific domains, *the gates*, have closed around the trapped substrate and ions. Despite the common steric occlusion of the substrate in all transporters, the degree to which different transporters block solvent accessibility to the binding pocket from the extracellular and cytoplasmic sides varies (Table 1). The independence of the gating process from the inward- to outward major conformation transition is emphasized by the observation that the occluded state might have either an outward- or an inward-facing direction, or for different transporters, we can conclude that there must be at least 5 structurally distinct conformations, as shown in Figure 2; outward-facing with extracellular gate open (cytoplasmic gate occluded), outward-facing with extracellular gate occluded (cytoplasmic gate remains occluded), intermediate with both gates occluded, inward-facing with gates occluded, inward-facing with only cytoplasmic gate open.

**Table 1:** **Transporters with known structures caught at various conformations**.

	**Substrate**	**Organism**	**Family**	**TMS Fold**	**Conformation**
**LacY**	Lactose H^+^ symporter	*E. coli*	MFS	6+6	-outward/occluded **[4OAA]**
					-inward/partially occluded (blocked with inhibitor) **[2Y5Y]**
					-inward/open **[2V8N, 2CFQ, 1PV7]**
**GlpT**	Glycerol-3-phosphate H^+^ symporter	*E. coli*	MFS	6+6	-inward/open **[1PW4]**
**FucP**	Fucose H^+^ symporter	*E. coli*	MFS	6+6	-outward/open **[3O7Q]**
**XylE**	Xylose H^+^ symporter	*E. coli*	MFS	6+6	-outward/partly occluded **[4GBY]**
**PepT**	Oligopeptide H^+^ symporter	*Streptococcus thermophiles* and *Shewanella oneidensis*	MFS	6+6	-occluded **[2XUT]**
					-inward/open **[4APS]**
**Glut1**	Glucose facilitator	*Human*	MFS	6+6	-inward-/open **[4PYP]**
**PiPT**	Phosphate transporter?	*Piriformospora indica* (fungus)	MFS	6+6	-inward/occluded **[4J05]**
**LeuT**	Amino acid Na^+ (*)^ symporter	*Aquifex aeolicus*	APC	5+5	-outward/open (blocked with Trp) **[3F3A]**
					-outward/occluded (blocked with inhibitors) **[2QEI], [2QJU]**
					-outward/occluded (inhibitor binding at secondary site on the gate) **[3GJD]**
					-outward/open (blocked with conformation-specific antibody) **[3TT1]**
					-inward-open (blocked with conformation-specific antibody) **[3TT1]**
**AdiC**	Arginine:agmatine antiporter		APC	5+5	Outward/open **[3OB6] [3LRB] [3NCY]**
					Outward/occluded **[3L1L]**
**vSGLT**	Galactose Na^+ (*)^ symporter	*Vibrio parahaemolyticus*	APC	5+5	-inward/occluded **[3DH4]**
					-inward/open **[2XQ2]**
**Glt*_PH_***	Glutamate transporter	*Pyrococcus horikoshii*	APC	5+5	-outward **[1XFH]**
					-intermediate **[3V8F]**
					-inward (cysteine cross-linking) **[3KBC]**
					-inward/open **[4P19]**
**UraA**	Uracil H^+ (*)^ symporter	*E. coli*	NAT/NCS2	7+7	-inward-/occluded **[3QE7]**
**Mhp1**	Benzyl-Hydantoin Na^+ (*)^ symporter	*Microbacterium liquefaciens*	NCS1	5+5	-outward **[2JLN]**
					-occluded **[2JLN]**
					-inward **[2X79]**
**DAT**	Dopamine Na^+^ symporter	*Drosophila melanogaster*	SLC	6+6	-outward/open (blocked with inhibitor) **[4M48]**
**CNT**	Uridine transporter	*Vibrio cholerae*	SLC	6+6	-inward/occluded **[3TIJ]**

The table includes the most prominent examples of transporter structures that helped in developing our ideas on how transport catalysis takes place at a molecular mechanistic level. All shown examples can be found in http://blanco.biomol.uci.edu/mpstruc/. The PDB code is included in the table. (

* *) denotes that these transporters are considered to be Na^+^ symporters based on crystallographic studies, but their prokaryotic origin suggest that they might well function as H^+^ symporters. MFS, Major Facilitator Superfamily; APC, Amino Acid-Polyamine-Organocation family; NAT/NCS2, Nucleobase/Ascorbate Transporter or Nucleobase:Cation Symporter-2 family; NCS1, Nucleobase:Cation Symporter-1 family; SLC, is the Solute carrier Transporter family. More information on transporter families is available in http://www.tcdb.org/*.

Thus, evidence supports the following sequence of events during transport catalysis. In an outward-facing conformation the extracellular gate fluctuates between occluded and open states allowing access of extracellular Na^+^ or H^+^. Binding of these cations, probably at the extracellular gate or deeper into the major substrate binding pocket, stabilizes the outward-facing open state, creating a high-affinity substrate-binding site. Following substrate and potentially additional ion binding, the extracellular gate closes and this closure promotes a transition to the intermediate or inward-facing occluded state. In contrast to the local changes associated opening and closing of gates, the isomerization between the outward- and inward-facing states involves larger-scale conformational changes spread throughout the transporter. This structural transition from outward- to inward-facing does not seem to involve the same domain movements in all transporters and further experimental and computational studies will be required to understand the movements associated with the transport cycle in each transporter studied. In the inward-facing state, the initial event triggering substrate release appears to be the release of Na^+^ or H^+^ ions. Following release of the symported cations, the open state of the intracellular gate is stabilized, leading to release of further ions and substrate. The empty inward-facing open transporter can then transit back outward-facing state and, thereby, complete the transport cycle. This model is predominantly based on the study of secondary active transporters and whether it is applicable to passive facilitators is still disputable (Naftalin, 2008, 2010).

As already highlighted above, recent evidence supports the idea that Na^+^ binding affects the opening and closing of the gates (Celik et al., 2008; Shi et al., 2008; Claxton et al., 2010; Krishnamurthy and Gouaux, 2012). But is there any evidence that substrates also have an effect on opening of gates? Are the gates themselves substrate-selective or they just open and close stochastically or in response to ion concentrations? To function as independent selective elements, the gates should act as transient secondary substrate binding sites. Alternatively, the gates might simply function as size- or charge-depended selective filters, when present in their open state. Recent ligand-binding experiments and molecular-dynamics simulations in LeuT, the *Aquifex aeolicus* amino acid/Na^+^ symporter which has been extensively used as a paradigm for understanding structure-function relationships in the Amino Acid-Polyamine-Organocation (APC) family, have suggested that there is an additional secondary binding site between the primary site and the bulk extracellular solution (Nyola et al., 2010; Thomas et al., 2014). Simultaneous occupancy of this secondary site seemed to trigger the intracellular release of substrate and sodium ions from the primary site. X-ray diffraction studies of LeuT, by contrast, do not show binding of either the substrate leucine or the substrate analog selenomethionine anywhere other than the primary binding site (Piscitelli et al., 2010; Penmatsa and Gouaux, 2014). On the other hand, LeuT complexed with tryptophan, which locks the transporter in an outward-open conformation, does bind a second tryptophan molecule in close proximity to the external gate (Singh et al., 2008). This site might be transiently occupied as substrates move from the extracellular vestibule to the primary binding site, when the transporter is in the outward-open conformation. In line with the existence of secondary binding sites in gates, some tricyclic antidepressants (TCAs), which have been reported to inhibit the human serotonin transporter SERT (an homolog of LeuT), were shown by docking studies, to bind in the predicted outward-facing gate, thus preventing further conformational changes needed for progress of the transport cycle (Gabrielsen et al., 2012). Moreover, crystal structures of a genetically engineered LeuT, resembling human biogenic amine transporter, showed that these inhibitors bind to the outward-facing gate and thus lock the transporter in an outward-facing open conformation (Wang et al., 2013). Related to these observations, TBOA (a bulky aspartate analog) binding in GltPh, an APC glutamate transporter from *Pyrococcus horikoshii*, blocks the closing of the external gate and inhibits the transporter (Crisman et al., 2009). The observation that bulky substrate analogs act as competitive inhibitors of transport by partially occupying the outward-facing gate of the transporter is also supported by substituted cysteine accessibility method (SCAM) assays for the eukaryotic NSS homolog GAT1 (Omoto et al., 2012), as well as the human glucose transporter hSGLT (Raja et al., 2012). Docking studies in Glut1, the human MFS type glucose facilitator, have also supported the existence of multiple substrate binding sites along a substrate translocation trajectory, some of which map close to putative outward- and inward-facing gates (Cunningham et al., 2006; Cunningham and Naftalin, 2013). Finally, flexible docking studies have shown a well-supported secondary binding site of substrates in the genetically identified putative external gate of UapA (Kosti et al., 2012). The importance of gates in substrate selection is thus more than apparent for the development of new rationally designed drugs.

## Activity-dependent control of transporter expression and turnover

Recent evidence strongly suggests that eukaryotic cells can directly “sense” the conformational status or the functioning of a transporter and accordingly regulate its turnover and possibly its membrane sorting. This idea is related to the discovery that several transporters are down-regulated by ubiquitination, endocytosis and vacuolar degradation in response to their transport activity. In other words, transporters are rapidly internalized from the plasma membrane (PM) and sorted to the endocytic/vacuolar pathway when cell grow in the presence of relevant substrates, but remain stable in the PM for many hours in the absence of external substrates. This phenomenon has been well studied in several fungal amino acid or purine transporters (Séron et al., 1999; Lin et al., 2008; Nikko et al., 2008; Nikko and Pelham, 2009; Gournas et al., 2010; Cain and Kaiser, 2011; Karachaliou et al., 2013; Crapeau et al., 2014), but also seems to occur in mammalians transporters involved in neurotransmission (Saunders et al., 2000; Whitworth and Quick, 2001; Chi and Reith, 2003; Melikian, 2004; Miranda and Sorkin, 2007; Zahniser and Sorkin, 2009; Okuda et al., 2011). Some yeast metal transporters are also down regulated either in the presence or in the absence of substrates, but in these cases turnover seems to be mostly via direct sorting from the Golgi to the endosomal/vacuolar pathway (Liu et al., 2007; Sullivan et al., 2007; Erpapazoglou et al., 2008).

The primary signal for substrate-elicited endocytic turnover of transporters is generally believed to be the over-accumulation of substrates at potential toxic levels. Transporter down-regulation is thought to provide a feedback control mechanism against cell poisoning form excess substrate. In this case, cellular levels of substrates would control the steady state levels of relevant transporter *indirectly*, through a yet unknown signaling pathway. However, recent evidence is against this scenario and rather strongly supports the idea that specific transporter conformations associated with an active transport cycle can be *directly* “read” by a mechanism, which elicits transporter ubiquitination and subsequently internalization and turnover. According to this model, the fate of a transporter at each transport cycle depends on its transition to an ubiquitination/endocytosis-eliciting conformation. In this case, a transporter can either perform a full transport cycle by going all the way to the inward-facing open conformation releasing the substrate, or trigger ubiquitination and endocytosis by persisting for sufficient time in an intermediate conformational state. More recent evidence suggests that the endocytosis-eliciting state is the substrate-occluded conformation (see below).

The above conclusions are mostly based on work performed with the UapA uric acid-xanthine transporter of *A. nidulans* (Gournas et al., 2010; Karachaliou et al., 2013) and two amino acid permeases, Gap1 and Can1, in *S. cerevisiae* (Cain and Kaiser, 2011; Schothorst et al., 2013; Ghaddar et al., 2014; Van Zeebroeck et al., 2014). In all three transporters, most inactive alleles or transporter versions with normal *K_m_* but very low *V_m_* values are blocked for substrate-elicited endocytosis. Moreover, in UapA, substrate analogs that are not transported (e.g., 3-methylxanthine) or internally accumulated substrates due to genetic mutations (e.g., uric acid accumulation in a null mutant of uric acid oxidase), do not elicit endocytosis. Finally, UapA or Can1 mutant versions with modified specificity undergo endocytosis in the presence of all “novel” substrates. For example, a UapA version (Gln408Glu/Thr526Leu) transporting efficiently hypoxanthine, in addition its natural substrates uric acid or xanthine, is endocytosed by all three purines (Gournas et al., 2010). Furthermore, a Can1 variant (Ser176Asn/Thr456Ser) converted to a lysine-specific permease is endocytosed by lysine instead of arginine (Ghaddar et al., 2014). These observations constituted primary evidence that transport-activity is absolutely necessary for endocytosis.

Interestingly however, in specific cases substrate-elicited endocytosis could be uncoupled from transport. In UapA, the hyperactive (increased transport rate) specificity mutant Phe528Ala, which transports with increased rate all purines, does not undergo substrate-elicited endocytosis. Similarly, in Can1, the hyperactive mutant Thr456Ser also did not undergo substrate-elicited endocytosis. In addition, in Can1, the inactivation of transport activity due to a mutation in a residue critical for proton-coupling (Glu184Gln) did not abolish substrate-elicited endocytosis. Uncoupling of transport-activity from endocytosis was also shown in Gap1 using different substrates or substrate analogs (Schothorst et al., 2013; Van Zeebroeck et al., 2014). In particular, L-Lysine is transported by Gap1 but, unlike most other substrates (e.g., all L-amino acids), does not trigger endocytosis. In addition, two transported, non-metabolizable analogs, β-alanine and D-histidine, are strong and weak elicitors of Gap1 endocytosis. Thus, mechanistically neither the completion of the transport cycle is a prerequisite for endocytosis, nor the block of the transporter cycle necessarily abolishes endocytosis. What seems to be necessary for endocytosis to occur is a specific intermediate transporter conformation, probably in a substrate-occluded state, persisting for a sufficient period of time. The acquisition of this intermediate transporter conformation might not be hindered by specific mutations, which however might be essential for subsequent steps necessary for transport. In this model, it is also reasonable to propose that the conformational change undergone by a transporter upon substrate binding would lead to a significant rearrangement on its cytosolic side and thus promote its recognition by the ubiquitination/endocytosis machinery. The model also suggests that to be efficiently endocytosed a transporter must remain “long enough” in the conformation recognized by this machinery.

Interestingly, some of the specificity mutations blocking substrate-elicited endocytosis in UapA, as for example Phe528Ser, map in the presumed external gate (Kosti et al., 2012). Notably, UapA-Phe528Ser is a fully functional transporter with normal *K_m_* values and increased transport rate, apparently due to the loosening of its external selective gate. This means that fine modifications solely in the gating mechanism can affect substrate-elicited endocytosis. This in turn suggests that effects in the gating mechanism have a downstream effect on the overall conformational dynamics of the transporter and the time persistence of specific intermediate conformations.

Among the transporters rigorously shown to undergo substrate-elicited endocytosis, Gap1 has been shown to have a second, transport-independent, function. In particular, Gap1 can transduce *signaling* of the PKA pathway in response to the presence external amino acids. This dual property of Gap1 has historically established the term *transceptor* for *trans*porters, which can also function as re*ceptors* involved in signal transduction pathways (Van Zeebroeck et al., 2009). In the case of Gap1, the sensing function could be uncoupled from the transport activity by screening for non-transporter amino acid analogs that elicit endocytosis, or transported analogs that do not trigger endocytosis (Van Zeebroeck et al., 2009; Schothorst et al., 2013; Van Zeebroeck et al., 2014). These observations indicate that signaling, similarly to endocytosis, requires a ligand-induced specific conformational change that may be part of, but does not necessarily require the completion of the transport cycle. Interestingly, endocytosis is not a prerequisite for signaling as these two phenomena can also be uncoupled (Van Zeebroeck et al., 2014).

Overall, results with UapA, Gap1, and Can1 support the concept that different substrates or ligands bind, probably to partially overlapping binding sites or with distinct orientations in a major substrate-binding pocket in transporters, triggering divergent conformational dynamics which eventually result in different conformation-induced downstream processes.

## Atypical transporter function: the case of allopurinol transport by UapA

The existence of gates in transporters does not violate the currently accepted model of the alternating access mechanism. It rather adds a further degree of complexity, related to how substrate specificity is determined by inter-domain synergy between the gates and a major binding site. However, alternative mechanisms on how transporters function have also been proposed. The most provocative of these models states that substrates “slide down” through several docking points in a channel-like trajectory, rather than inducing abrupt alternating conformational changes exposing a single major substrate binding site extra- or intracellularly (Naftalin, 2008, 2010). This model is mostly supported through docking studies and thermodynamic approaches performed with specific facilitators, but also gains genetic support in cases where mutations mapping outside the major substrate-binding site modify substrate specificity. In addition, in some cases some transporters do not seem to follow the typical Michaelis-Menten kinetic behavior, compatible with a single substrate-binding site. This review does not intend to extensively describe cases of transporter atypical behavior (for this see the review Conde et al., 2010). It rather attempts to highlight selected cases that might provoke, at least in specific cases, the re-evaluation of the mechanism of transporter action in relation to drug uptake or efflux. One such case concerns the uptake of allopurinol by *A. nidulans*.

Allopurinol is a structural isomer of hypoxanthine (4-hydroxy[3,4-d]-pyrimidine) which acts as substrate and non-competitive inhibitor of xanthine oxidase/dehydrogenase (XO/XDH), a key enzyme for purine catabolism (Lewis et al., 1978; Pacher et al., 2006). Through its inhibitory activity on XO/XDH, allopurinol blocks uric acid production and is thus used for gout treatment (reviewed in Lipkowitz, 2012). Importantly, allopurinol has been used to treat *Leishmania* infections and also displays activity against the related parasite *Trypanosoma brucei* (de Koning and Jarvis, 1997; Natto et al., 2005; Mishra et al., 2007). In contrast to other antiprotozoan drugs, which are associated with severe side effects and emerging resistance, allopurinol either alone or in combination with other drugs, has proved to be more effective against cutaneous infections. Consequently, transporters specific for allopurinol have been identified at the molecular level in *Leishmania* and *Trypanosoma* species. All known protozoan transporters are high affinity (μM range) H^+^ symporters and exhibit a rather broad specificity for most purines, pyrimidines and several analogs (de Koning and Jarvis, 1997; Al-Salabi et al., 2003; Burchmore et al., 2003; de Koning et al., 2004; Al-Salabi and de Koning, 2005; Natto et al., 2005). Interestingly, allopurinol has not been used as an antifungal or antibacterial agent. In fungi, allopurinol uptake has only been studied in *A. nidulans*, where genetic and physiological evidence confirmed that UapA is the major gateway for high-affinity, low-capacity, allopurinol uptake (Scazzocchio et al., 1973; Diallinas and Scazzocchio, 1989). Interestingly however, in the course of identifying the transport mechanism of allopurinol transport, two unexpected kinetic observations became apparent; first, UapA-mediated radiolabeled allopurinol transport is non-saturable and H^+^ gradient independent, and secondly, the uptake of radiolabeled xanthine, rather than being inhibited, as expected, by excess allopurinol, it is stimulated (Diallinas, 2013). The effect of allopurinol on xanthine uptake was not reciprocal, as excess xanthine or other substrates of UapA inhibit allopurinol uptake. In addition, flexible docking approaches failed to detect allopurinol binding in the major UapA substrate binding site. Although the stimulating effect of allopurinol on xanthine transport could, in principle, be rationalized by a cryptic allopurinol-specific allosteric site, evidence was obtained supporting that accelerated influx of xanthine is triggered through exchange with cytoplasmically accumulated allopurinol. Further kinetic experiments strongly suggested that allopurinol is transported by facilitated diffusion through a substrate translocation trajectory, which is distinct from, but probably overlapping with, that of physiological UapA substrates (Diallinas, 2013).

The atypical phenomenon underlying allopurinol transport by UapA was rationalized by the existence of substrate-specific alternative mechanisms and partially overlapping translocation trajectories in a single transporter. Interestingly, the existence of alternative and partially overlapping substrate trajectories has also been shown in the mammalian glucose transporter GLUT1 (Jiang et al., 2010). The message from these studies is that transporter-dependent cellular uptake or efflux of drugs might not show the typical kinetic behavior expected, and in these cases more rigorous biochemical or genetic approaches should be performed to understand the mechanism behind the phenomenon. On the other hand, the atypical behavior of a transporter might also become an advantage, especially when combination or cocktails of drugs is used, as the presence of a substrate might stimulate the translocation of another, the latter being the one with cytotoxic activity.

## Conclusions

Transporters, despite their proven role in drug uptake and efflux, have been little exploited toward this direction, obviously due to the significant lack of knowledge concerning their function, specificity and regulation of expression. Currently nearly 70 *bona fidae* transporter crystal structures are known, of which less than 50 concern secondary active transporters, facilitators or antiporters, and only 4 of them are of eukaryotic origin. Bacterial transporter structures are used quite successfully to model human homologs, but this approach risks erroneous or overstated conclusions. This is so because bacterial transporters are usually smaller in length due to much shorter N- or C- terminal regions, or internal hydrophilic loops, which in eukaryotes have been shown to be extremely critical for transporter function, specificity, cellular sorting and turnover. In addition, several of the known prokaryotic structures correspond to transporters of unknown physiological function, specificity and regulation, simply because these proteins proved more stable for crystallography.

The lack of knowledge on transporters, especially those of eukaryotes, is obviously due to general technical difficulties associated with their transmembrane nature, but also due to limited use of appropriate genetic or other alternative approaches to structural biology. In this review, I selected to present some paradigmatic genetic studies on fungal transporters, which led and continue to lead to novel knowledge on transporter function and regulation. In my opinion, one of the most prominent recent discoveries on transporter function is the existence of channel-like gates. Their existence was indeed predicted using rigorous genetic and biochemical approaches, before crystals became available and confirmed their presence. A second novel finding is related to substrate-elicited endocytosis and in particular to the idea that eukaryotic cells directly sense transporter function and accordingly regulate their turnover in order to achieve cell homeostasis to specific physiological, developmental or stress conditions. Impressively, what has been found on fungal transporter substrate-elicited endocytosis seems to hold true for mammalian transporters involved in neurotransmission. Finally, I gave an example, using a fungal transporter, but also discussing a similar case in a human transporter, on how transporters can, in some cases, show atypical kinetic behavior.

In summary, I think we need to learn much more on transporter function and regulation before we use them as tools in drug action. Recent crystal structures of mostly prokaryotic transporters and recent functional and computational studies of eukaryotic homologs have generated hypotheses on how transporters accomplish solute uptake. However, not all states in the transporter cycles have yet been identified in transporters, and only 4 crystal structures are available for eukaryotic transporters. More crystal structures might help in solving these controversies. In addition, many of the states proposed from existing crystal structures still need to be verified by other methods. To obtain new knowledge we definitely need to use appropriate eukaryotic model systems and interdisciplinary approaches, including functional genomics, reverse genetics, easy transport assays, *in vivo* microscopy, rational and random genetic screens, in addition to crystallography and other biophysical approaches. Knowledge of the different states in the transporter cycles at a molecular level will eventually assist in developing new highly targeted drugs.

### Conflict of interest statement

The author declares that the research was conducted in the absence of any commercial or financial relationships that could be construed as a potential conflict of interest.
